# Low HDL Cholesterol, Smoking and IL-13 R130Q Polymorphism are Associated with Myocardial Infarction in Greek Cypriot Males. A Pilot Study 

**DOI:** 10.2174/1874192400802010052

**Published:** 2008-07-22

**Authors:** Stavroulla Xenophontos, Marilena Hadjivassiliou, Alexandros Karagrigoriou, Nafsika Demetriou, George Miltiadous, Ioannis Marcou, Moses Elisaf, Dimitri P Mikhailidis, Marios A Cariolou

**Affiliations:** 1 Department of Cardiovascular Genetics & The Laboratory of Forensic Genetics, The Cyprus Institute of Neurology and Genetics, Nicosia, Cyprus; 2 Mathematics and Statistics Department, University of Cyprus, Nicosia, Cyprus; 3 Department of Internal Medicine, Medical School, University of Ioannina, Ioannina, Greece; 4 Department of Internal Medicine, Nicosia General Hospital, Nicosia, Cyprus; 5 Department of Clinical Biochemistry, Royal Free Hospital Campus, University College London, Pond Street, London, NW3 2 QG, UK

**Keywords:** Smoking, myocardial infarction, cholesterol, Cyprus

## Abstract

This study was carried out in Greek Cypriot males to identify risk factors that predispose to myocardial infarction (MI). Genetic and lipid risk factors were investigated for the first time in a Greek Cypriot male case-control study.****Contrary to other studies, mean low density lipoprotein cholesterol did not differ between cases and controls. High density lipoprotein cholesterol on the other hand, although within normal range in cases and controls, was significantly higher in the control population. In agreement with many other studies, smoking was significantly more prevalent in cases compared with controls. In pooled cases and controls, smokers had a significantly lower HDL-C level compared with non-smokers. The frequency of the IL-13 R130Q homozygotes for the mutation (QQ), as well as the mutant allele were significantly higher in cases compared with controls. The IL-13 R130Q variant, or another locus, linked to it, may increase the risk of MI.

## INTRODUCTION

Identified modifiable cardiovascular disease (CVD) risk factors include dyslipidaemia, hypertension, diabetes, smoking, obesity and metabolic syndrome [1-6]. Dyslipidaemia is a major risk factor for CVD and improving lipid levels with statins is an effective regimen [7-1]. Many genetic risk factors have also been identified which are pro-thrombotic, pro-inflammatory or pro-atherogenic [12,13]. It is also important to consider gene-gene interactions and gene-environment interactions since the combination of risk factors may increase the severity of CVD and the risk of morbidity and mortality [14]. In the present study, a battery of functional gene polymorphisms that have been reported to increase the risk for myocardial infarction (MI) was selected for investigation. The following genetic variables were tested: IL6-174G/C, IL13 R130Q, Stromelysin 5A/6A, ACE D/I, ApoE, GPIIIa A1/A2, MTHFR C677T, ECNOS G894T, Factor V G1691A, Factor II G20210A, PAI-1 4G/5G and PON1 L55M. Cases (n = 77) and controls (n = 79) were unrelated individuals, age 30-65 years.

Two of the most intensively studied candidate gene polymorphisms are the ACE D/I and Apo E gene polymorphisms. The former plays a role in blood pressure (BP) regulation while the latter influences lipid metabolism. Several studies found an association between the deletion allele of the ACE gene and the E4 allele of the ApoE gene and CVD [15, 16]. In contrast, case-control studies in the Greek population do not support the latter association of CVD with the Apo E4 allele and further, a protective role was reported for the E3/4 genotype, as well as the E2 allele [17-19]. The paraoxonase 1 (PON1) L55M gene polymorphism was investigated because this is an integral enzyme of the high density lipoprotein cholesterol (HDL-C) molecule that can protect low density lipoprotein cholesterol (LDL-C) from oxidative modification and subsequent initiation and progression of atherosclerosis [20]. Factor V, factor II, glycoprotein IIb/IIIa (GPIIb/IIIa) and plasminogen activator inhibitor type 1 (PAI-1) gene polymorphisms were also investigated. These are functional prothrombotic polymorphisms and have been implicated in the pathogenesis of MI [21-23]. The Endothelial constitutive nitric oxide synthase gene (ecNOS), G894T polymorphism was investigated as an association with the T allele and CVD was found in some studies [24-27]. The MTHFR C677T mutation has been studied to clarify its role in CVD. This mutation in the homozygous state is associated with raised circulating total homocysteine levels, possibly predisposing to CVD [28]. In contrast others showed no association between the MTHFR C677T mutation and CVD [29]. Matrix metalloproteinases are involved in plaque rupture. The stromelysin gene (MMP-3) exhibits a 5A/6A polymorphism where the 5A allele, is associated with susceptibility to MI [30].

Increasing evidence implicates inflammation in atherosclerosis and ischaemic heart disease (IHD) and in particular pro-inflammatory cytokines may promote thrombosis [31, 32, 12]. An imbalance between pro-inflammatory and anti-inflammatory cytokines (i.e. a predominance of Th1 over Th2 cytokines) is thought to play a pivotal role in the development of IHD [33-37]. We chose interleukin-6 (IL-6), which has both pro- and anti-inflammatory properties and has been investigated in relation to MI. One study showed that the C allele of the IL-6 –174G/C promoter polymorphism was associated with MI and another reported raised IL-6 levels in MI patients [38, 39]. Interleukin-13 (IL-13) is a Th2 cytokine with anti-inflammatory properties which has been implicated in the pathogenesis of asthma [40]. This cytokine has not been investigated with respect to CVD but evidence from a study in patients with asthma revealed that women but not men were at increased risk of developing IHD and this could possibly be linked to IL-13 [41]. Indeed several IL-13 polymorphisms were identified which are associated with increased levels of IgE, a predictor of asthma phenotype [42]. The functional polymorphism, IL-13 Arg130Gln, was associated with raised IgE in asthma patients and this was selected in our study [40].

## MATERIALS AND METHODOLOGY

### Characteristics of the Cohort

Cases (n = 77) and controls (n = 79) were unrelated individuals aged 30-65. Impaired renal function, hypothyroidism or abnormal liver functions were exclusion criteria. None of the patients recruited were on hypolipidaemic or antihypertensive drug therapy. They were all residents of the same area; a southern coast city (Larnaca, Cyprus). Cases were individuals, who had experienced a non-fatal first MI. The diagnostic criteria were 2 of the following 3 observations: an increase in creatine kinase (CK-MB) activity, an abnormal electrocardiogram and intense chest pain of long duration (WHO diagnostic criteria). The MI patients were recruited for this study 2 days after their MI. Questionnaires were completed; lipid profiles were measured within the first 18 h after the MI event. Blood samples were collected for DNA extraction. A resting BP measurement was taken and body mass index (BMI) was calculated. The family history was also recorded. All subjects who participated in this study gave their informed consent, and all experimental procedures were carried out in accordance with the ethical standards of the local ethics committee and with the Helsinki Declaration of 1975, as revised in 2000.

## DNA ANALYSES

Genotyping for the various polymorphisms was performed as previously described [20-24, 28, 43, 44] with the exception of the IL-13 Arg130Gln polymorphism which was genotyped using a novel allelic discrimination TaqMan assay designed at the Department of Cardiovascular Genetics & Laboratory of Forensic Genetics, Cyprus Institute of Neurology and Genetics, Nicosia, Cyprus. The assay was run on an ABI Prism 7700 Sequence Detector (Foster City, USA). The primers and probes were designed using specified software (Primer Express Version 1.5, Foster City, USA), which takes into consideration the optimum conditions for allelic discrimination. The amplicon was 78 bp long. The following PCR primers and allelic discrimination probes were designed and used for genotyping:

Forward PCR primer – AGGACCTGCTCTTACATTTAAAGAAACT,

Reverse PCR primer – TGCAAATAATGATGCTTTCGAAGT,

Allele 1 /probe 1- wild type (VIC – AGGGAC**G**GTTCAACT, amino acid residue-arginine-R),

Allele 2 /probe 2- mutated (FAM – AGGGAC**A**GTTCAACT, amino acid residue-glutamine-Q),

The assay was validated on 20 samples of known genotype using a previously designed PCR assay with the following primers: 5'-CTT CCG TGA GGA CTG AAT GAG ACG GTC-3' (forward) and 5'-GCA AAT AAT GAT GCT TTC GAA GTT TCA GTG GA-3' (reverse), followed by *Nla*IV restriction digestion [40]. The procedure for TaqMan Assay performance and data analysis was carried out as instructed by the manufacturer of the ABI Prism 7700 Sequence Detector. PCR reagents (UNIVERSAL MASTER MIX) and oligo-nucleotides (PRIMERS & VIC/FAM LABELED PROBES) were purchased from Applied Biosystems (Foster City, USA). In brief, the PCR conditions for the TaqMan assay were as follows: 50^0^C - 2 min x 1 cycle (Temperature at which UNG is activated to degrade any PCR product that may have contaminated sample, as dUTP is used in place of dTTP for PCR in TaqMan assay), this is followed by 95^0^C - 10 min (AmpliTaq Gold DNA polymerase activation) x 1 cycle, 95^0^C - 15 secs (denaturation) then 60^0^C - 1 min (Annealing & Extension) x 40 cycles.

## STATISTICAL ANALYSES

The statistical package SPSS V12 was used to perform the relevant statistical tests. Briefly, comparison of frequencies of genotypes and alleles were achieved using the chi-square test. Distribution of quantitative variables was determined using the Kolmogorov-Smirnov test. Mean values of quantitative variables between groups were compared using the unpaired t-test for normal data and Mann-Whitney for non-parametric data. Bivariate correlation analysis was carried out to determine any correlation between pairs of variables (Pearson’s correlation). Binary logistic regression analysis was performed to determine the relative contribution of IL-13 Arg130Glu polymorphism, lower HDL-C and smoking on the dependant variable MI.

## RESULTS

The frequency of diabetes in cases and controls was 16% and 19%, respectively and for dyslipidaemias these values were 56% and 54%, respectively. There were no significant differences when proportions were compared (chi-square analysis). Mean age between male cases and controls was not significantly different (54 ± 8 in cases vs 52 ± 10 years in controls). BMI and BP were all normally distributed. No significant differences were observed between cases and controls (Table **1**). Total and HDL-C were normally distributed, triglycerides were positively skewed but LDL-C was normally distributed. Mean values for total, LDL-C and HDL-C were compared between cases and controls using the unpaired t-test and for triglycerides values were compared using the Mann-Whitney test. Mean values for triglycerides, total and LDL-C did not show any significant differences. A significantly higher mean HDL-C level was observed in controls compared with cases (45 ± 12 mg/dl vs 40 ± 9 mg/dl; P = 0.001) (Table **1**).

In the case group, 83% were smokers, whereas in controls only 58% were smokers (Chi-square test P = 0.001). Mean HDL-C levels were significantly higher in all non-smokers compared with all smokers (46 ± 13 mg/dl vs 41 ± 9 mg/dl; P = 0.013). A significantly higher mean value HDL-C was observed in controls who did not smoke compared with controls who did smoke (49 ± 14 mg/dl vs 45 ± 10 mg/dl; P = 0.038). In cases, mean HDL-C level did not differ between smokers and non-smokers (40 ± 9 mg/dl vs 39 ± 9 mg/dl; P = 0.759). HDL-C levels were significantly higher in controls who did not smoke compared with the corresponding MI group (49 ± 14 mg/dl vs 39 ± 9 mg/dl; P = 0.029) (Table **2**).

Cross tabulations between genotype and allele frequencies in cases and controls indicated that frequencies were homogeneous between the 2 groups for all polymorphic loci tested except for the IL-13 R130Q polymorphism. All genotypes were in Hardy-Weinberg equilibrium in cases and controls (see Table **3**). For the IL-13 R130Q polymorphism, the frequency of heterozygotes (RQ) as well as homozygotes for the mutation (QQ) was significantly higher in cases compared with controls, (0.40 vs 0.28 for RQ and 0.09 vs 0.02 for QQ; P = 0.030). In addition, the difference in the mutant allele frequency was significant, (0.29 in cases vs 0.16 in controls; P = 0.011). Data are shown in Table **4**.

The correlation matrix between all variables involved was constructed for easy comparison between correlations and for determining clusters of variables that covary. The elements (coefficients) of the matrix are used not only as measures of the degree and direction between row and column variables and the proportion of covariation but also as the guide to the logistic multiple regression statistical analysis that follows. The significant correlations are listed in Table **5**. The******categorical independent variable smoking and the categorical dependent variable MI were categorized as “1” if the answer was “yes” i.e. that individual did smoke and he had an MI; and as “2” if the reverse was true, i.e. that individual did not smoke and he did not have an MI. By performing the correlation analysis between these 2 variables, MI was positively correlated with a positive smoking habit (r = 0.270, P = 0.001). HDL-C level was positively correlated with the absence of an MI event (r = 0.263, P = 0.001). The IL-13 R130Q gene polymorphism was given the following codes in the data base: “1-RR; 2-RQ; 3-QQ”. An inverse correlation was observed between the IL-13 R130Q gene polymorphism and the absence of an MI event (r = -0.212, P = 0.008). Smoking w as inversely correlated with HDL-C level (r = -0.201, P = 0.013) (Table **5**).

Contrary to most other reports, in this particular male population, there was no correlation between ApoE genotype and any of the lipid variables (Pearson correlation analysis) nor was there any significant difference between mean lipid levels when stratified by ApoE genotype using ANOVA. However, a trend was observed which was consistent with the established effect of the ApoE genotype on lipid levels. Specifically, mean total cholesterol level was 214 ± 40 mg/dl in individuals who had the 2/3 ApoE genotype (n=20), 223 ± 47 mg/dl in individuals who had the 3/3 ApoE genotype (n=115), and 231 ± 55 mg/dl in individuals who had the 3/4 ApoE genotype (n=16). The same trend was observed for LDL-C while triglycerides and HDL-C were more homogeneous for the 3 genotypes.******Again there were no significant differences.

Variables that were significantly different between cases and controls in earlier statistical analyses were selected for binary logistic regression analysis. These were HDL-C, smoking and the IL-13 R130Q polymorphism. This analysis kept the IL-13 R130Q polymorphism, low HDL-C and smoking status as predictors of MI occurrence. For smoking, if an individual smoked the odds of an MI occurring were 2.7 times more likely than if an individual did not smoke (P = 0.016). For HDL-C as the concentration increased the odds of an MI occurring decreased, specifically, as the HDL-C level increases by 1 mg/dL, the odds of an MI occurring decreased by 5.4%. (P = 0.006). Finally, an individual who was an IL-13 QQ homozygote was found to be 5.8 times more likely to experience an MI than one with the wild type genotype; RR (P = 0.039) and an individual with an RQ genotype was 2.1 times more likely to do so (P = 0.055). Data are shown in Table **6**. If the analysis is repeated with variables: smoking, hypertension, hypercholesterolaemia, diabetes, HDL cholesterol and the IL13 R130Q SNP, the results indicate that, smoking, HDL cholesterol and the IL13 R130Q SNP are still significant risk factors for MI, whereas hypertension, hypercholesterolaemia and diabetes are not, as indicated previously in t-tests and correlation analysis.

## DISCUSSION

Smoking and a comparatively low HDL-C level were associated with MI in Greek Cypriot men. The term “comparatively low” for HDL-C in our study actually refers to a value that is at the borderline of the acceptable lower limit of the normal range as defined by a pan–European survey of 8,545 dyslipidaemic patients where an HDL-C <40 mg/dL in men is defined as low [45]. Smoking has been shown to be independently associated with vascular disease and to interact with other genetic and environmental risk factors in the causation of vascular damage [46, 47]. More specifically, the pathogenic effects of smoking are attributed to its ability to increase LDL-C, plasma triglycerides and VLDL triglycerides and to simultaneously lower HDL-C [48]. Furthermore, smoking has been shown to increase oxidation and nitration of LDL-C which promote the atherogenic process [49, 50]. Since a high percentage of the case group are smokers then it is highly likely that some of the above proath-erogenic mechanisms may have contributed to the occurrence of MI. Our observations are in agreement with those of other CHD case-control studies [51, 52]. However, several other common CHD risk factors did not show an association in this study, while a novel CHD predictor emerged, the IL-13 R130Q polymorphism. Due to the limited number of participants in the present pilot study, this interesting association needs to be confirmed in larger studies and the possible effect of other linked loci to the IL13 gene excluded.

Surprisingly, the established risk factor LDL-C was homogeneous between cases and controls and mean values were within normal range. Although mean LDL-C was homogeneous between the 2 groups, LDL subfraction distribution may have been heterogeneous, being more pro-atherogenic (LDL-3 to 7) in the case group and therefore potentially contributing to the risk of MI [53-55]. Survivors of MI may have an abundance of small, dense LDL in plasma compared with controls and further, that patients who also smoked had a more atherogenic LDL subfraction profile [56]. Small, dense LDL particles are more atherogenic than large buoyant LDL particles because they are more susceptible to form oxidized LDL and are not readily cleared [57]. Furthermore, since the immediate pre-MI, LDL-cholesterol levels were not available, we cannot rule out the possibility that prior to MI these values were higher than after the MI event. Triglycerides levels have been reported to increase small, dense LDL particles and therefore the risk of cardiovascular disease [58, 59]. In the present study, however, a significant difference is not observed. The range in controls was greater than that of cases due to the presence of an outlier (who smoked 40-50 cigarettes per day) in the control group with 800 mg/dL triglycerides. The exclusion of this outlier from the analysis did not alter the distribution of these data or result in a significant difference in triglyceride levels between the 2 groups. Since there is no difference between groups in triglyceride levels, the hypothesized difference in the LDL subfraction profile in our 2 groups may be mediated by other factors such as smoking [58-60]. In contrast to the above hypotheses, no correlation was observed between smoking and triglyceride level in this study, or between MI occurrence and triglyceride level. It is therefore more likely that any effects of the hypothetical proatherogenic small dense LDL profile preponderance in cases compared to controls is caused largely by smoking.

Future studies will need to include LDL subfractions. Such testing has in fact been suggested by the (NCEP ATP III) National Cholesterol Education Program Adult Treatment Panel in addition to the conventional lipid profile [61]. In addition, the ApoE genotype did not seem to have a significant effect on total cholesterol level. The Mediterranean diet may account for the relatively normal mean cholesterol level in the study population as a whole.

A significantly higher mean HDL-C level was observed in controls compared with cases. In cases, the mean HDL-C level was low irrespective of smoking status suggesting that other factors may be involved in lowering this protective factor. Other genetic factors or dietary habits/deficiency may be the cause of the significantly lower HDL-C in cases compared with controls. HDL-C levels were significantly higher in controls that did not smoke compared with the corresponding MI group (48 mg/dl vs 39 mg/dl P = 0.029). The latter comparison highlights the effect of low HDL-C *per se* on the occurrence of an MI. Our findings regarding HDL-C suggest that the currently accepted lower value of (40 mg/dl) as satisfactory****should be raised (Adult Treatment Panel III-www.nhlbi.nih.gov/guidelines/cholesterol, 2005). However, such a suggestion is limited by the small numbers in our study and it may only apply to a Mediterranean population known to have a very high smoking rate. Several intervention studies, such as the Veterans Affairs High-Density Lipoprotein Cholesterol Intervention Trial (VA-HIT) and HDL-Atherosclerosis Treatment Study (HATS), provided evidence that a rise in HDL-C significantly reduces cardiovascular risk [6-8, 62-64]. The protective effect of HDL arises from multiple actions including reverse cholesterol transport [63, 65, 66]. Furthermore, HDL enhances nitric oxide synthesis and improves endothelium-dependent relaxation [67].

Many studies focused on lipid lowering as a way of reducing the risk for CVD. However, with recent evidence for the involvement of inflammation in atherosclerosis, it is relevant that statins exert anti-inflammatory effects. This effect together with lipid lowering and other actions may contribute to the overall clinical benefit observed in trials [32, 68, 69].

The association of the IL-13 R130Q polymorphism with MI in Greek Cypriot men in the present pilot study is quite novel and provides the impetus for the design of future experiments using a larger cohort size. The investigation of this variant at different stages of acute coronary syndromes (ACS) may also be an important study, as the variant may be of predictive value for MI and may have important implications for preventive and therapeutic regimens. In light of the many functions of IL-13 and the pathways in which it is involved, some hypotheses may be proposed regarding this novel association. Wild type IL-13 suppresses macrophage production of proinflammatory mediators, regulates extracellular matrix, inhibits tissue factor expression induced by bacterial lipopolysaccharides, reduces the pyrogenic effects of IL-1 or TNF thus protecting endothelial and monocyte surfaces against inflammatory mediator-induced procoagulant changes [70-72]. It is possible, therefore that this variant may, favour coagulation, thrombus formation and coronary artery occlusion or it may activate matrix metalloproteinases and consequently extracellular matrix degradation leading to coronary artery plaque destabilization, rupture and ultimately MI. Furthermore, a comparison of recombinant wild type IL-13 and IL-13 R130Q activity in primary monocytes has indicated that IL-13 R130Q is more active in inducing the main steps in the IL-13-dependent signaling pathway, including IgE synthesis [73]. In relation to this, an earlier study reported that among other raised inflammatory markers encountered in MI patients, raised IgE is also observed. It is further suggested that this may participate in plaque-rupture and ultimately in MI events [74]. An, alternative hypothesis is that these studies also provide a possible mechanism which may explain the association of the IL-13 R130Q mutation, (possibly by raising IgE) and occurrence of MI events in our pilot study. In future studies we will investigate whether MI patients will show a concurrent association with the IL13 R130Q mutation, raised IL-13 activity as well as raised IgE. Additionally, the IL-13 R130Q variant has been linked to eosinophilia, and the latter has been shown to predict cardiovascular and cerebrovascular mortality possibly through endothelial inflammation and ultimately atherosclerosis. The mechanisms suggested include the secretion of 2 proteins (cationic protein and major basic protein) which activate mast cells to release histamine which causes coronary artery spasm and arrhythmias. Secondly, arachidonate 15-lipoxygenase which is expressed at high levels in eosinophils may be involved in oxidative modification of LDL cholesterol and therefore in atherosclerosis, a major cause for MI [75, 76]. In conclusion, the important findings of this first pilot study concerning Greek Cypriot men and MI, point out that larger scale studies should be encouraged to confirm and study further the role of the putative novel genetic risk factor IL-13 R130Q and lipid levels in MI in Cyprus.

## Figures and Tables

**Table 1.** Characteristics of Cases and Controls

**Variable Mean (± SD) or Frequency **	**Cases n = 77**	**Controls n = 79**
Age	54 (± 8)	52 (± 10)
P value	0.224	
Frequency of diabetes	16%	19%
P value	0.600	
Frequency of dyslipidaemia	56%	54%
P value	0.845	
Frequency of Hypertension	28%	24%
P value	0.611	
Frequency of Smoking	83%	58%
P value	0.001	
BMI	28.2 (± 4)	28.0 (± 4)
P value	0.764	
Total Cholesterol (mg/dl)	220 (± 45)	225 (± 48)
P value	0.460	
LDL-C (mg/dl)	139 (± 42)	142 (± 40)
P value	0.615	
HDL-C (mg/dl)	40 (± 9)	45 (± 12)
P value	0.001	
Triglycerides* * (mg/dl)	156(53-480)	160 (23-800)
P value	0.728	

P- value for t-test (comparison of means). BMI = body mass index; LDL-C = low density lipoprotein cholesterol; HDL-C = high density lipoprotein cholesterol;* * Median value and range in parenthesis, comparison of medians by non-parametric statistics (Mann-Whitney test).

**Table 2.** Comparison of Mean High Density Lipoprotein Cholesterol (HDL-C) Levels (mg/dl) in Cases and Controls as A Function of Smoking Status

	Mean HDL-C Level	P-Value
	(± SD)	T-Test
Cases	40	0.001
Smokers + Non	(± 9)	
(77) smokers		
Controls	46	
Smokers + Non	(± 12)	
(79) smokers		
Cases + Controls	41	0.013
Smokers (109)	(± 9)	
Cases + Controls	46	
Non-smokers (46)	(± 13)	
Controls Smokers	45	0.038
-45	(± 9)	
Controls	49	
Non-smokers (33)	(± 14)	
Cases	40	0.759
Smokers (64)	(± 9)	
Cases	39	
Non-smokers (13)	(± 9)	
Controls	48	0.029
Non-smokers (33)	(± 14)	
Cases	39	
Non-smokers (13)	(± 9)	

Value in brackets indicates sample size.

**Table 3.** Rare Allele Frequencies and 95% Confidence Intervals for Gene Polymorphisms in Male Cohort

**Gene Polymorphism**	**Rare Allele**	**Relative Frequency (95%CI) Cases**	**Relative Frequency (95%CI) Controls**	**P-value**
IL6 -174G/C	C	0.20 (0.14-0.25)	0.22 (0.15-0.28)	0.356
IL13 R130Q	Q	0.29 (0.21-0.34)	0.16 (0.11-0.23)	0.011^+^
Stromelysin 5A/6A	5A	0.42 (0.35-0.49)	0.34 (0.27-0.42)	0.172
ACE D/I	I	0.33 (0.25-0.38)	0.34 (0.26-0.41)	0.719
ApoE (Hinf1)*	2	0.05 (0.03-0.08)	0.08 (0.06-0.11)	0.487
ApoE (Hinf1)*	4	0.06 (0.04-0.09)	0.05 (0.03-0.08)	
GPIIIa A1/A2	A2	0.18 (0.12-0.23)	0.14 (0.09-0.20)	0.512
MTHFR C677T	T	0.40 (0.32-0.47)	0.36 (0.29-0.44)	0.642
ECNOSG894T	T	0.28 (0.23-0.37)	0.31 (0.24-0.38)	0.937
FactorV G1691A	A	0.04 (0.03-0.11)	0.07 (0.03-0.12)	0.938
Factor II G20210A	A	0.03 (0.01-0.06)	0.03 (0.00-0.05)	0.913
PAI-1 4G/5G	4G	0.41 (0.33-0.48)	0.49 (0.41-0.56)	0.167
PON1 L55M	M	0.40 (0.32-0.48)	0.46 (0.38-0.53)	0.106

* ApoE Hinf1 polymorphism has three alleles 2/3/4; ApoE alleles 2 and 4 are considered rare alleles; +Difference in allele frequencies between cases and controls is statistically significant.

**Table 4.** IL-13 R130Q Genotype and Allele Frequencies in Case & Control Groups (Numbers in Parenthesis Represent Relative Frequencies)

**Group**	**IL-13R130Q**					**Total N**
	**Genotypes**			**Alleles**		
	**RR**	**RQ**	**QQ**	**R**	**Q**	
Cases	39 (0.51)	31 (0.40)	7 (0.09)	109 (0.71)	45 (0.29)	77
Controls	55 (0.70)	22 (0.28)	2 (0.02)	132 (0.84)	26 (0.16)	79
P-value	0.030			0.011		

Pearson’s chi square test to compare frequency of genotypes and alleles of the; IL-13R130Q polymorphism between cases and controls.

**Table 5.** Pearson’s Correlation Coefficients and P-Values for Bivariate Correlation Analyses

Pairs of Variables	Pearson Correlation	P-value
Correlated	Coefficient	
MI-SMOKING	0.27	0.001
NO MI-HDL-C	0.263	0.001
MI-IL-13R130Q	-0.212	0.008
SMOKING-HDL-C	-0.201	0.013

MI = myocardial infarction; HDL-C = high density lipoprotein cholesterol; IL-13 = interleukin 13. The categorical independent variable smoking and the categorical dependent variable MI were categorized as “1” if the answer was “yes” i.e. that individual did smoke and he had an MI; and as “2” if the reverse was true, i.e. that individual did not smoke and he did not have an MI. MI was positively correlated with a positive smoking habit.HDL-C level was positively correlated with the absence of an MI event. The IL-13 R130Q gene polymorphism was given the following codes in the data base: “1-RR; 2-RQ; 3-QQ”. An inverse correlation was observed between the IL-13 R130Q gene polymorphism and the absence of an MI event. Smoking was also inversely correlated with HDL-C levels.

**Table 6.** Beta-Lactamase Assay Protocol for Trk-NFAT-bla CHO-K1 Cells in 384-Well Format

**Variable**	**B**	**S.E.**	**Wald**	**df**	**Sig.**	**Exp(B)**	**95,0% C.I. for EXP(B)**	
							**Lower**	**Upper**
Smoker	0.981	0.407	5.796	1	0.016	2.667	1.200	5.925
HDL-C	-0.056	0.020	7.662	1	0.006	0.946	0.909	0.984
IL-13			6.804	2	0.033			
IL-13(1-RQ)	0.723	0.378	3.671	1	0.055	2.061	0.984	4.320
IL-13(2-QQ)	1.758	0.851	4.263	1	0.039	5.799	1.093	30.754
Constant	1.237	0.924	1.792	1	0.181	3.444		

HDL-C = high density lipoprotein cholesterol; IL-13 = interleukin 13. Variables which are significant contributors to the occurrence of an MI in a sample of Cypriot men (smoking, relatively low HDL-C and the IL-13 R130Q mutation).
